# Cell migration and proliferation are regulated by miR-26a in colorectal cancer via the PTEN–AKT axis

**DOI:** 10.1186/s12935-019-0802-5

**Published:** 2019-04-02

**Authors:** Jossimar Coronel-Hernández, Eduardo López-Urrutia, Carlos Contreras-Romero, Izamary Delgado-Waldo, Gabriela Figueroa-González, Alma D. Campos-Parra, Rebeca Salgado-García, Antonio Martínez-Gutierrez, Miguel Rodríguez-Morales, Nadia Jacobo-Herrera, Luis Ignacio Terrazas, Abraham Silva-Carmona, César López-Camarillo, Carlos Pérez-Plasencia

**Affiliations:** 1 Laboratorio de Genómica Funcional, Unidad de Biomedicina, FES-IZTACALA, UNAM, Tlalnepantla, Mexico; 2 Laboratorio de Genómica, Instituto Nacional de Cancerología, Av. San Fernando No 22, Col. Sección XVI, Tlalpan, Zip code 14080 Mexico City, DF Mexico; 3 Unidad de Bioquímica, Instituto de Ciencias Médicas y Nutrición, Salvador Zubirán, Tlalpan, Mexico City, DF Mexico; 4 Laboratorio de Inmunología de Parásitos, Unidad de Biomedicina, FES-IZTACALA, UNAM, Tlalnepantla, Mexico; 5 Laboratorio de Genética, Genómica y Bioinformática, Hospital Infantil de México, Mexico City, Mexico; 6 Posgrado en Ciencias Genómicas, Universidad Autónoma de la Ciudad de México, Mexico City, Mexico; 7 Programa de Doctorado en Ciencias Biomédicas, Universidad Nacional Autónoma de México, Mexico City, Mexico

**Keywords:** MicroRNA, mir-26a, PTEN, AKT, Colorectal cancer, Animal model for carcinogenesis

## Abstract

**Background:**

Invasion and metastasis are determinant events in the prognosis of Colorectal cancer (CRC), a common neoplasm worldwide. An important factor for metastasis is the acquired capacity of the cell to proliferate and invade adjacent tissues. In this paper, we explored the role of micro-RNA-26a in the regulation of proliferation and migration in CRC-derived cells through the negative regulation of PTEN, a key negative regulator of the AKT pathway.

**Methods:**

Expression levels of PTEN and mir-26a were surveyed in normal and CRC-derived cell lines; paraffin embedded human tissues, TCGA CRC expression data and a Balb/c mice orthotopic induced CRC model. CRC was induced by an initial intraperitoneal dose of the colonic carcinogen Azoxymethane followed by inflammatory promoter Dextran Sulfate Sodium Salt. Luciferase assays provide information about miR-26a–PTEN 3′UTR interaction. Proliferation and migration by real time cell analysis and wound-healing functional analyses were performed to assess the participation of mir-26a on important hallmarks of CRC and its regulation on the PTEN gene.

**Results:**

We observed a negative correlation between PTEN and mir-26a expression in cell lines, human tissues, TCGA data, and tissues derived from the CRC mouse model. Moreover, we showed that negative regulation of PTEN exerted by miR-26a affected AKT phosphorylation levels directly. Functional assays showed that mir-26a directly down-regulates PTEN, and that mir-26a over-expressing cells had higher proliferation and migration rates.

**Conclusions:**

All this data proposes an important role of mir-26a as an oncomir in the progression and invasion of CRC. Our data suggested that mir-26a could be used as a biomarker of tumor development in CRC patients, however more studies must be conducted to establish its clinical role.

## Background

Colorectal cancer (CRC) is the third most common neoplasm and the fourth cause of cancer-related death worldwide in both sexes. Invasion and metastasis are determinant events in the prognosis of CRC. An important factor for metastasis is the acquired capacity of the cell to proliferate and invade adjacent tissues. One of the most relevant signaling pathways regulating cell proliferation, survival, angiogenesis, and metastasis is PI3K/AKT; which is negatively regulated by the tumor suppressor Phosphatase and Tensin homolog (PTEN) [1]. Loss of PTEN function occurs in several types of cancer—including CRC—through various genetic mechanisms such as point mutations or allelic loss of chromosome 10q2; however, biallelic inactivation of this site has not been demonstrated. Finally, methylation of the PTEN promoter has been reported to be associated in high microsatellite instability in 19% of colorectal cancers [2] and the PTEN messenger has been demonstrated to be targeted by microRNA regulation [3].

Micro-RNAs (miRNAs) are non-coding short RNAs that modulate gene expression by inducing mRNA degradation or translational repression [4]. They perform this function by binding to the 3′ UTR of their target mRNA through complete or partial base complementarity, thus they are capable of pleiotropic effects [5, 6]. Deregulation of the expression patterns of several microRNAs has been implicated in establishment and progression of many types of cancer. Particularly, mir-26a has been associated to development of glioblastoma [7], cholangiocarcinoma [8] and ovarian cancer [9] and thus labeled as an oncomir in those cancers; however, mir-26a has also been classified as tumor suppressor in pancreatic cancer [10], hepatocellular carcinoma [11] and nasopharyngeal carcinoma [12].

In CRC, miR-26a is significantly upregulated [13], but the function and clinical relevance of this miRNA in CRC is still partially understood. Our group has recently found that in CRC Rb1 gene is a target of miR-26a [14], but this is still far from the complete picture, as a single miRNA has been observed to target several genes. For example, miR-182-5p targets three genes involved in DNA repair [15], and drives metastasis of primary sarcomas [16]. On the other hand, various miRNAs can target the same gene, yielding similar effects in spite of being different regulators; hence, overexpression of both mir-130a [17] and mir-23a [18] enhance migration, invasion and the epithelial-mesenchymal transition (EMT) in osteosarcoma cells through direct PTEN regulation.

The aim of present study was to further explore the participation of miR-26a in CRC development, through the analysis of the relationship between mir-26a expression and PTEN. We found that miR-26a does regulate PTEN, abrogating its expression both in CRC-derived cell lines and in a mouse model that closely resembles colitis-mediated CRC. Moreover, over-expression of mir-26a mimic increases the phosphorylation levels of AKT T-308 that is the active form of AKT and triggers cell migration, proliferation among other hallmarks of cancer. Our findings suggest that miR-26a is indeed a key regulator of colorectal carcinogenesis since it targets at least two important tumor suppressor genes Rb and PTEN; thus, miR-26a is also a promising molecular biomarker involved in the progression of colon carcinogenesis process.

## Methods

### Patient samples

Twenty CRC paraffin-embedded tissue samples staged locally advanced; ten Crohn’s disease paraffin-embedded tissue samples and 13 healthy tissues from colorectal were obtained by colonoscopy without macro and microscopic lesions from Instituto Nacional de Cancerología—National Cancer Institute, Mexico pathology registry, present investigation was approved by ethics committee (approval number INCAN/CI/826/17). None of the authors had access to potentially identifying information from the donors of the paraffin-embedded samples.

### Tissue expression for in silico meta-analysis

Mature Mir-26a (RefSeq MI0000083) and mRNA PTEN (NM_001304717) expression data were obtained from The Cancer Genome Atlas in different stages of 424 (75 Stage I, 304 Stage II-III and 45 Stage IV) Colorectal Cancer samples and were normalized with deseq 2 (Bioconductor Package; data were compared with 41 healthy tissues. To assess the protein expression of PTEN; antibody-based proteomic data was obtained from The Human Protein Atlas [19]; staining intensity was compared between all the available healthy and cancer tissue data (2 and 13, respectively) from the PTEN entry. miR26a is encoded in two *loci*; mir-26a1 is localized in chromosome 3 within the CTDSPL gene and mir-26a2 is in chromosome 12 within the CTDSP2 gene.

### Cell culture and transfection

CRC-derived HCT116 cells (ATCC CCL-247) were cultured in RPMI medium supplemented with 10% (v/v) fetal bovine serum and maintained (FBS) at 37 °C with 5% CO2. CRC-derived SW480 (ATCC CCL-228), SW620 (ATCC CCL-227) and non-tumoral immortalized epithelial CRL1790 colon cells obtained from ATCC were cultured in DMEM F12 medium supplemented with 10% (v/v) fetal bovine serum and maintained at 37 °C with 5% CO_2_.

All employed plasmids were transfected using Lipofectamine 2000 transfection agent (Invitrogen), following the manufacturer’s protocol. Mirvana Micro-RNA mimics and inhibitors (Ambion) were transfected using the siPORT NeoFX transfection agent (Life Technologies) following the manufacturer’s protocol. Unless otherwise indicated, RNA and protein expression was analyzed 24 h post-transfection.

### CRC mouse model

Twelve female Balb/c mice (Harlan Laboratories, México) aged 6 weeks used in this study were maintained at Facultad de Estudios Superiores Iztacala Animal Facility according to the institutional animal care guidelines (number of protocol FES-2016-1423). Animals were housed in plastic cages (6 mice/cage) with drinking water and pelleted basal diet ad libitum under controlled humidity (50 ± 10%), light (12/12 h light/dark cycles) and temperature (23 ± 2 °C). CRC was induced by an initial intraperitoneal dose of the colonic carcinogen Azoxymethane (AOM) followed by three Dextran Sulfate Sodium Salt (DSS) 7d-long, ad libitum administrations during the second, 5th and 8 weeks of treatment. This mouse model was thoroughly described in a previous paper from our group [20].

Three randomly chosen mice were euthanized after each DSS dose for mRNA and protein analysis, so as to have three biological replicates of every mRNA or protein expression measurements. Large bowels were flushed with saline and excised. Inflammation-related cancer development was confirmed by histological analysis.

### RNA expression analysis

Large bowel parts of each experimental group mice were homogenized by triplicate in a Bullet Blender (Next Advance) following the manufacturer’s protocol for intestinal tissue. Total RNA was isolated from the homogenized samples or from cultured cells (CRL1790, HCT116, SW480 or SW620) grown to approximately 80–85% confluence, using the TRIzol reagent (Invitrogen) following the manufacturer’s protocol. miRNAs were isolated from tissue blocks using the miRNeasy FFPE kit (Qiagen) following the manufacturer’s recommendations.

Mature miR-26a and the PTEN messenger were detected in the murine model samples by RT-PCR using a Roche Light Cycler 2.0. For miR-26a, cDNA was generated from 100 ng total RNA with the TaqMan Micro-RNA Reverse Transcrtiption Kit (Applied Biosystems) in a 15 µL volume; qPCR was performed using 1 µL cDNA and the mir-26a taqman probe (Applied Biosystems). Amplification conditions were 10 min at 95 °C, followed by 40 cycles of 95 °C for 15 s, 68 °C for 60 s. For PTEN mRNA detection, we used Titan One RT-PCR kit (Roche) supplemented with SybrGreen and the following primers: Fw AGGCACAAGAGGCCCTAGAT, Rv AACTGAGGATTGCAAGTTCCG. cDNA was synthesized at 50 °C for 30 min, immediately followed by denaturation at 94 °C for 2 min, 40 cycles of 94 °C for 10 s, primer-dependent annealing temperature for 30 s and 68 °C for 45 s, and a final extension at 68 °C for 7 min.

MiR-26a and the PTEN messenger were detected in cultured cells and tissue block samples by RT-PCR using the Bio-Rad CFX 96 Touch and the mir-26a taqman probe (Applied Biosystems) or the SYBR Select Master Mix for CFX (Applied Biosystems). Amplification conditions for miR-26a were as mentioned above. For PTEN mRNA detection, cDNA was synthesized from 2 µg total RNA using the High-Capacity cDNA Reverse Transcription Kit (Roche); a twentieth of this reaction was used for qPCR. Amplification conditions were 2 min at 95 °C for initial denaturation, followed by 40 cycles of 95 °C for 15 s, primer-dependent annealing temperature for 15 s and 72 °C for 60 s.

Relative expression data was calculated through the ΔΔCt method (Applied Biosystems) and normalized relative to U6 snRNA or GAPDH mRNA accordingly.

### Protein expression analysis

Protein extracts from large bowel parts of each experimental mouse group of from cultured cells was obtained by homogenization in RIPA buffer (SantaCruz Biotechnology); a Bullet Blender (Next Advance) and stainless-steel beads was used for bowel tissues. Protein extract was cleared by centrifugation at 12,000 rpm for 20 min.

For inmmunodetection, 50 µg total protein from tumor tissue or cultured cells were mixed with Laemmli sample buffer, boiled, separated in 12% or 15% SDS-PAGE and transferred onto a Hybond-P PVDF membrane (Amersham-GE Healthcare). Membranes were probed overnight using a 1:500 (v/v) dilution of the anti-PTEN (Sc-7974) and AKT total (Sc-H-136) (Santa Cryz Biotechnologies, CA, USA) and phosphorylated (AKT-phospho-T308, Ab-38449) (Abcam, Cambridge, UK); for detection, 1:2500 (v/v) dilutions of HRP anti-rabbit or anti- mouse conjugate antibodies (SantaCruz Biotechnology) were used. Finally, using the SuperSignal WestFemto chemiluminescent substrate (Thermo Scientific), the membranes were scanned in the C-Digit blot scanner (Li-Cor) and the images were analyzed for densitometry in the associated ImageStudio software (LiCor). Membranes were stripped and re-probed for detection of actin (anti-actin, Sc-47778) as a loading control. A representative image from three independent experiments is shown.

### Luciferase reporter assays

Reporter plasmids were constructed by ligation of synthetic oligonucleotide duplexes (IDT) containing putative miR-26a target regions in the PTEN 3′UTR: 5′- CTA GTT AAC TGT TAG GGA ATT TTA CTT GAA A -3′ and 5′-AGC TTT TCA AGT AAA ATT CCC TAA CAG TTA A-3′, obtained from microRNA.org [21] to form a DNA duplex with overhanging SpeI and HindIII half sites in the 5′ and 3′ ends respectively, which was cloned into the appropriately digested pMIR-REPORT plasmid (Ambion). This construct was co-transfected with miR-26a mirVana miRNA mimic (Applied Biosystems) and the pMIR-REPORT β-gal Control Plasmid (Ambion) into HCT116 cells. Luciferease activity was analyzed using the Dual-Luciferase Reporter Assay System (Promega) 48 h after transfection, in a GloMax 96 Microplate Luminometer (Promega). Luciferase activity was normalized to β-gal activity for each transfected well; each experiment was performed in triplicate.

### Real-time analysis of cell proliferation and migration

The xCELLingence real-time cell analyzer (RTCA) instrument was used with E-plates to analyze proliferation and with CIM-plates (ACEA, Biosciences) to monitor migration of cells transfected with miR-26 mimic and inhibitor. For proliferation assays, HCT116 cells were cultured and transfected in 6 well-plates (5 × 105 cells per well) with 10% FBS-supplemented medium at 37 °C for 24 h, after that cells were trypsinized and counted by Neubauer chamber. We plated 1 × 104 cells per E-plate well with 10% FBS-supplemented in 150 µL/well. The RTCA recorded cell index values over 24 h by 15 min-intervals. For migration assays, 1 × 104 cells per well were cultured in 150 µL without FBS-avoiding therefore, cell proliferation- in the upper CIM-plate chamber while 160 µL/well of 10% FBS-supplemented medium was added as a chemoattractant to the lower chamber. The cell index values recorded by 15 min-intervals over 24 h. These experiments were performed by triplicate.

### Wound healing assay

A wound healing assay was performed to assess migration. HCT116 cells were seeded into 6 well-plates (4 × 105 cells per well) and were maintained with 2% FBS-supplemented medium to avoid cell proliferation at 37 °C for 24 h. These cells were transfected with mir-26a mimic, anti-mir26a or controls using Lipofectamine 2000 as transfection reagent for 6 h. Afterwards, the medium was removed and replaced with freshly-changed medium 2% FBS-supplemented medium, and a wound was performed with a sterile 200 µL pipette tip in each well. Cells were monitored every 24 h for 72 h.

### Statistical analysis

All values are expressed as the mean ± SEM. Data were analyzed in the Prism 5.0 (GraphPad) software using a one-way ANOVA analysis followed by Tukey’s Multiple Comparison Test.

## Results

### PTEN and mir-26a expression are inversely correlated in human CRC samples

**Fig. 1:**
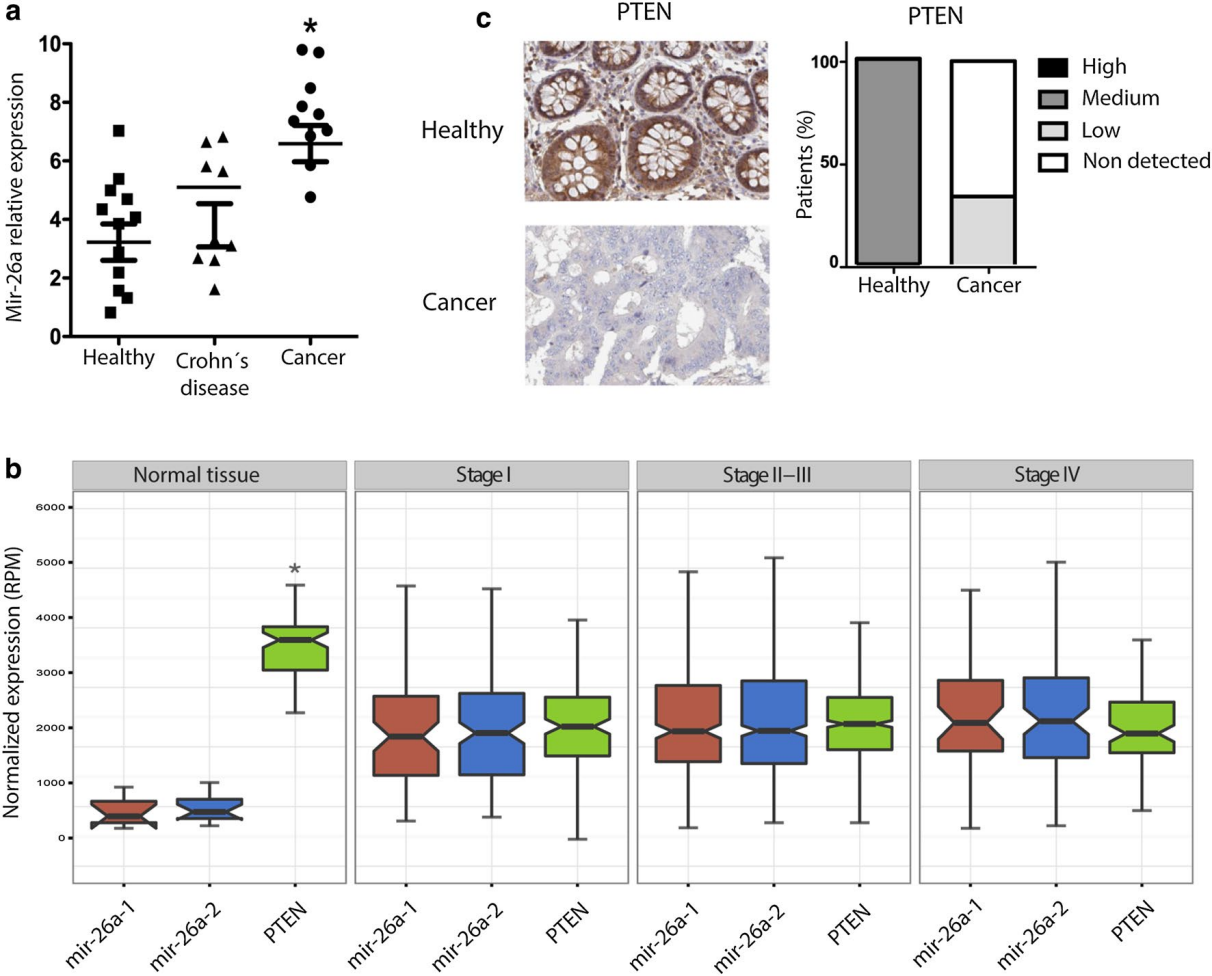
*PTEN is down*-*regulated in CRC human samples*. **a** mir-26a expression in CRC-derived paraffin-embedded normalized with RNU6 and compared with Crohn´s disease samples as control (*p < 0.05). **b** mir-26a and PTEN expression data in different stages of CRC obtained from “The Cancer Genome Atlas” (*p < 0.05). **c** Percent of PTEN antibody staining in CRC and healthy samples obtained from “The Human Protein Atlas”

We measured mir-26a expression in CRC-derived paraffin-embedded tissue samples and found heterogeneous expression of this microRNA. The average mir-26a tumor expression was significantly higher than that found in both Crohn’s disease and healthy tissue samples suggesting that mir-26a was overexpressed in CRC tissue samples (Fig. 1a). To verify whether this pattern was consistent in a broader sample, we used data from a third source—The Cancer Genome Atlas—and analyzed expression levels of PTEN and mir26a in 41 normal samples: 75 Stage I, 304 Stage II-III and 45 Stage IV (Fig. 1b). The data showed that mir-26a was significantly upregulated in tumors compared to normal tissues; although there was no significant difference between expression in different CRC stages. Correspondingly, PTEN was downregulated in tumors, further confirming the inverse correlation between mir-26a and PTEN that we had observed in paraffin embedded tissues samples results. Respecting to PTEN protein levels in situ we performed an in silico analysis sourcing data from the Human Protein Atlas database and compared the protein expression levels of PTEN in 2 healthy and 13 CRC samples. Immunohistochemistry data showed positive PTEN staining in all healthy samples, but none in CRC samples, indicating that loss of PTEN expression has an inverse correlation with mir-26a overexpression in CRC samples (Fig. 1c; compare to 1a and b).

### PTEN is downregulated in CRC-derived cell lines

**Fig. 2:**
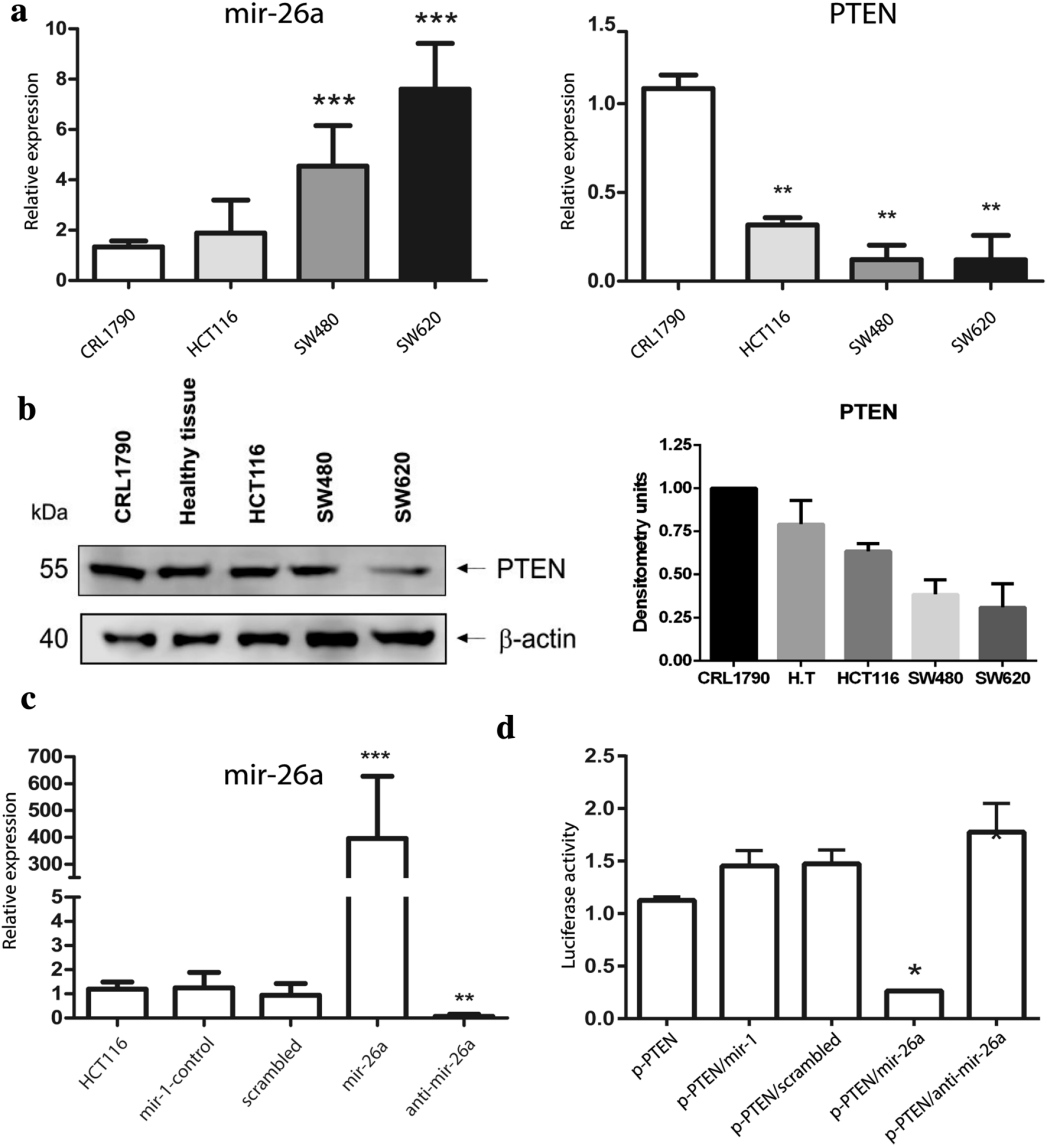
PTEN is under-expressed in CRC cell lines through mir-26a regulation. **a** Expression of PTEN and miR-26a in CRL1790 (control), HCT116, SW480 and SW620 (CRC cell lines) normalized with GAPDH or RNU6, respectively. **b** Western blot was performed to detect the expression of PTEN in the different cell lines and a mouse healthy tissue (H.T), b-actin was used as loading control. **c** mir-26a was transfected in HCT116 cell line and was measured by real-time, mir-26a expression was normalized with RNU6 PCR. **d** Luciferase reporter assay using mir-26a/PTEN interaction region in HCT116 cell lines normalized with empty vector

We measured the expression of mir-26a and PTEN in HCT116, SW480 and SW620, corresponding to stages I, III and metastatic respectively by qRT-PCR; CRL1790 non-tumoral colon cells were used as control. As shown in Fig. 2a, we found a slight increase in the expression level of mir-26a in HCT116; however, in SW480 and SW620 cell lines we observed a high expression of this microRNA, four- and seven-fold respectively. We consistently found an inverse correlation between mRNA PTEN and miR-26a levels: relative expression of PTEN was significantly decreased in every CRC cell line. Nevertheless, PTEN mRNA levels were diminished in HCT116 and this cell line did not have changes in mir-26a expression, suggesting there is another mechanism that regulates PTEN levels in HCT116 or Stage 1 of CRC. In all cell lines tested and mouse healthy tissue, PTEN protein levels were concordant to RNA levels. Besides, PTEN protein detection reflected an inverse correlation with miR-26a levels (Fig. 2b). Together, these results showed that both mRNA and protein levels of PTEN are downregulated and correlate with mir-26a overexpression in CRC-derived cell lines suggesting a possible regulation of this miRNA.

### mir-26a directly inhibited PTEN expression through 3′ UTR interaction

To confirm whether mir-26a directly regulates the PTEN mRNA, we used a reporter construct harboring the 3′ UTR specific binding site sourced from Targetscan bioinformatics algorithm (microRNA.org) downstream from the Luciferase gene to form p-Luc-PTEN. From the previously assayed cell lines, HCT116 showed better transfection capability and the lowest endogenous mir-26a expression, so we employed them to assess the negative regulation of PTEN exerted by mir-26a. HCT116 cells were transfected with mir-26a mimic; then the expression level of miR-26 was measured by qRT-PCR to standardize transfection conditions (Fig. 2c); later, we co-transfected p-Luc-PTEN with mir-26a for 48 h, which resulted in 74% reduction in luciferase levels compared to empty vector. These findings indicated that mir-26a could bind the 3′ UTR of the PTEN mRNA (Fig. 2d).

### mir-26a regulates PTEN expression in a CRC-derived cell line

**Fig. 3:**
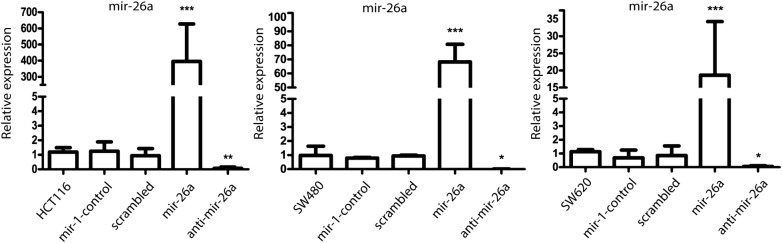
Transfection of mir-26a mimic or inhibitor is detected by RT-PCR in colon cancer cell lines. Successful transfection of mir-26a and controls was detected by means of qRT-PCR in HCT116, SW480 and SW620

**Fig. 4:**
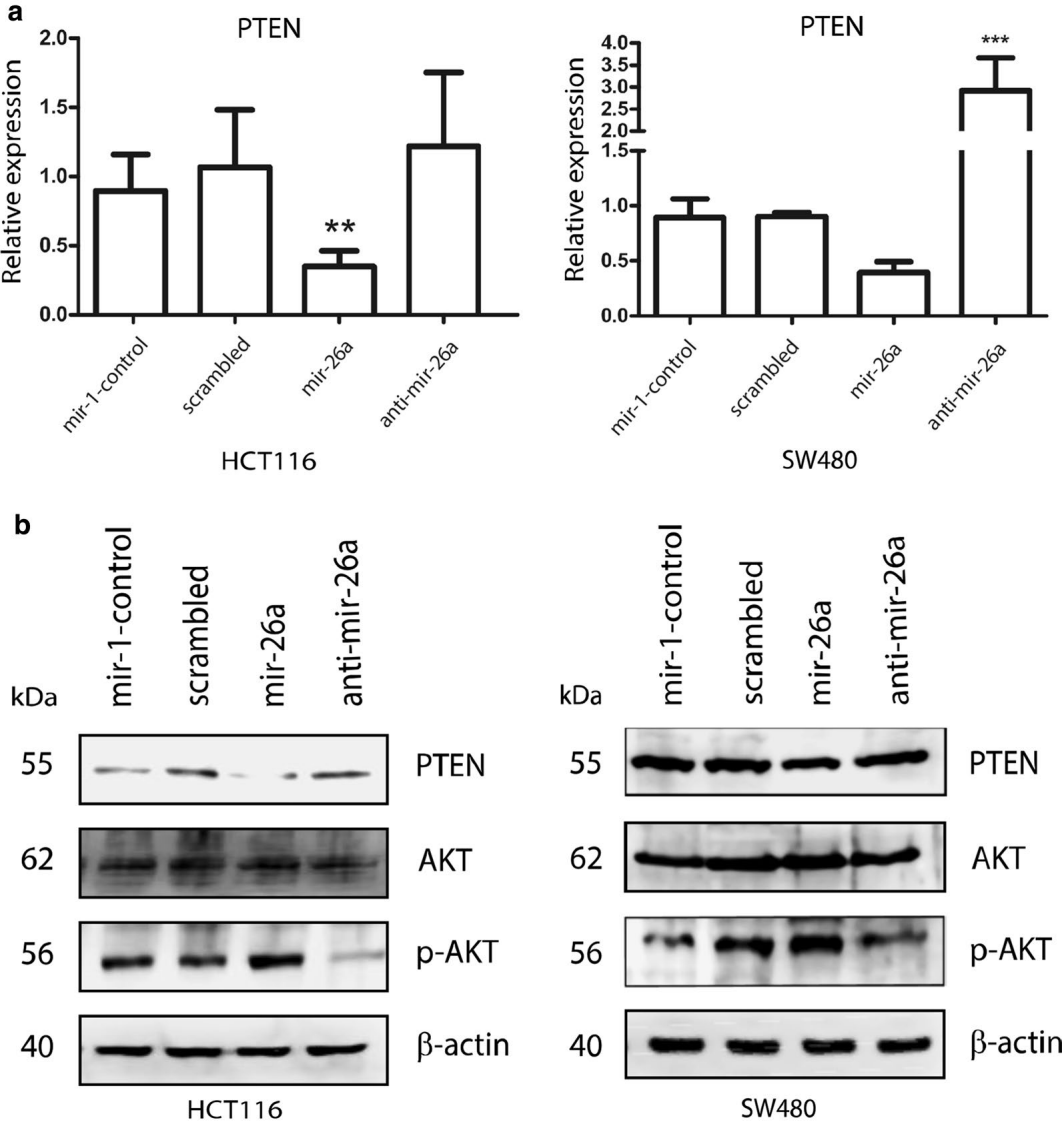
Overexpression of mir-26a diminished PTEN mRNA and protein. HCT116 and SW480 cell lines were transfected with mir-1-control, scramble sequence, mir-26a mimic and mir-26a inhibitor, the expression of PTEN was measured by real-time PCR and western blot at 48 h post-transfection. **a** mRNA expression and **b** protein detection normalized with GAPDH and b-actin respectively. PTEN was diminished in presence of mir-26a

To clarify the effect of mir-26a on PTEN expression, mir-26a was overexpressed or repressed in HCT116, SW480 and SW620 CRC cell lines by transfection with a mir-26a mimic or inhibitor. qRT-PCR detection of miR-26a in these cells and the corresponding controls, confirmed successful transfection (Fig. 3). We observed a better transfection capability in HCT116 and SW480 cell lines, so we chose them for following experiments. PTEN mRNA was measured by qRT-PCR and it was decreased by miR-26a over-expression in both cell lines (Fig. 4a); however, a slight increase was observed with anti-miR-26 in HCT116, whereas a substantial increase was found in SW480. Finally, the same results were observed at the protein level in PTEN, while AKT did not show evident changes at protein level; however, we observed a direct correlation between mir-26a expression and p-AKT (Thr308)—the active state of AKT—in both cell lines (Fig. 4b), indicating that mir-26a affects PTEN expression and AKT activity in CRC cells.

### mir-26a regulates proliferation and migration in HCT116 cells

**Fig. 5:**
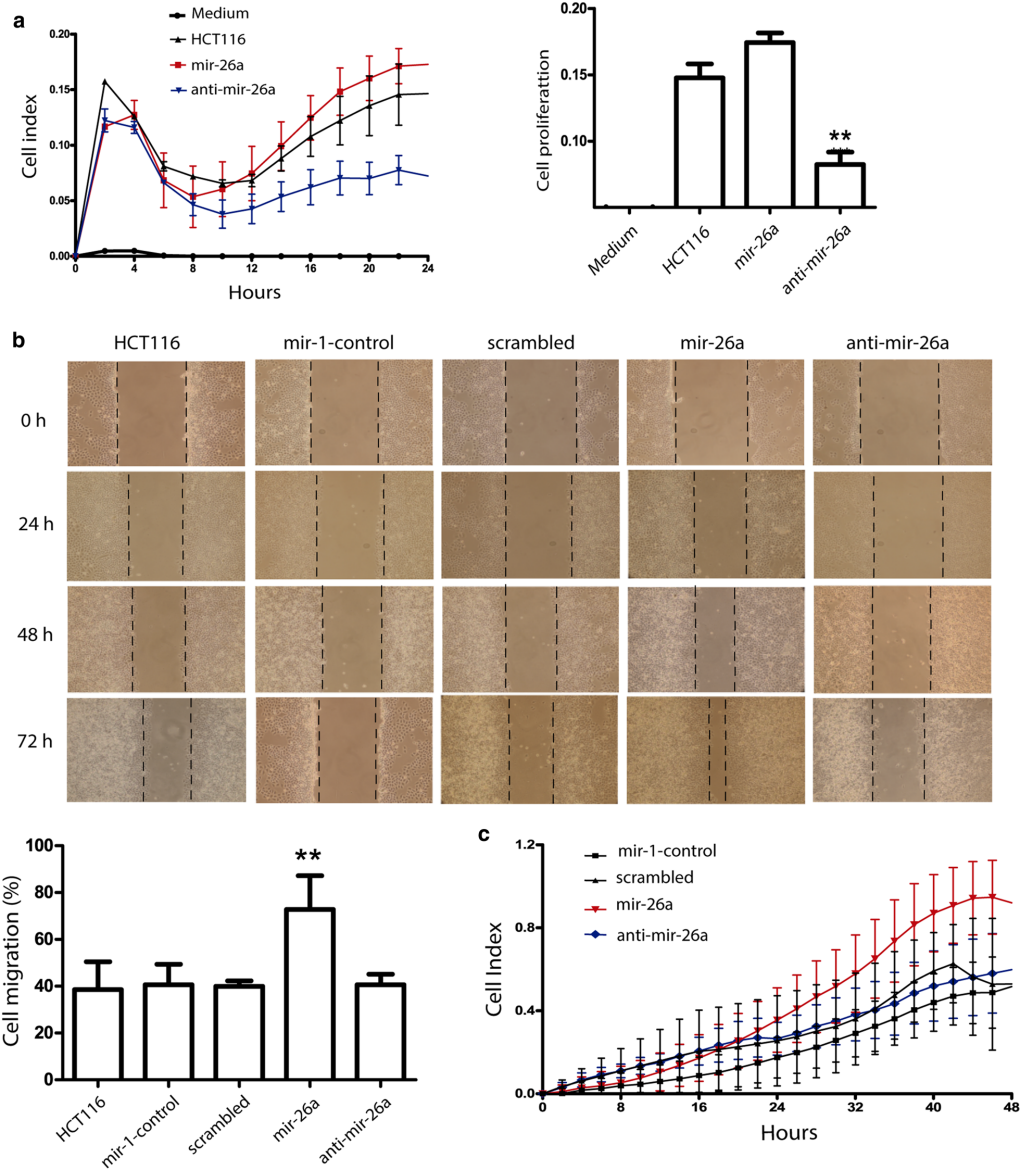
mir-26a over-expression enhance migration and its inhibition stops HCT116 cell proliferation. HCT116 cell line was transfected, mir-26a mimic or mir-26a inhibitor and **a** proliferation was measured by xCELLingance system in HCT116 cell line at 24 h. On other hand **b** migration was measured by Scratch assay in HCT116 cell lines at 72 h (HCT116 cells were cultured with 2% FBS medium to minimize cell proliferation) and it also was measured by **c** RTCA xCELLingance system at 48 h. mir-26a enhanced proliferation and migration

PTEN is involved in the negative regulation of AKT activation, which affects cell proliferation and migration among other crucial hallmarks of cancer. We used the xCELLingence RTCA system to measure cell proliferation and migration in each group. As expected, mir-26a overexpression increased slightly cell proliferation, on the other hand, the mir-26a downregulation, significantly decreased the proliferation rate by > 50% compared to untransfected HCT116 cells indicating that mir-26a does not increase proliferation but its presence is pivotal to keep it (Fig. 5a). Next, we examined the role of mir-26a in CRC cell migration using the xCELLingence CIM plate and wound healing assays. We observed that cell migration was significantly increased after transfection with mir-26a mimic in both experiments (33%) compared to their respective controls (Fig. 5b, c). These observations suggest that mir-26a plays an important role in the proliferation maintenance and promote migration process in CRC.

### mir-26a is overexpressed in AOM/DSS-induced CRC mouse model

**Fig. 6:**
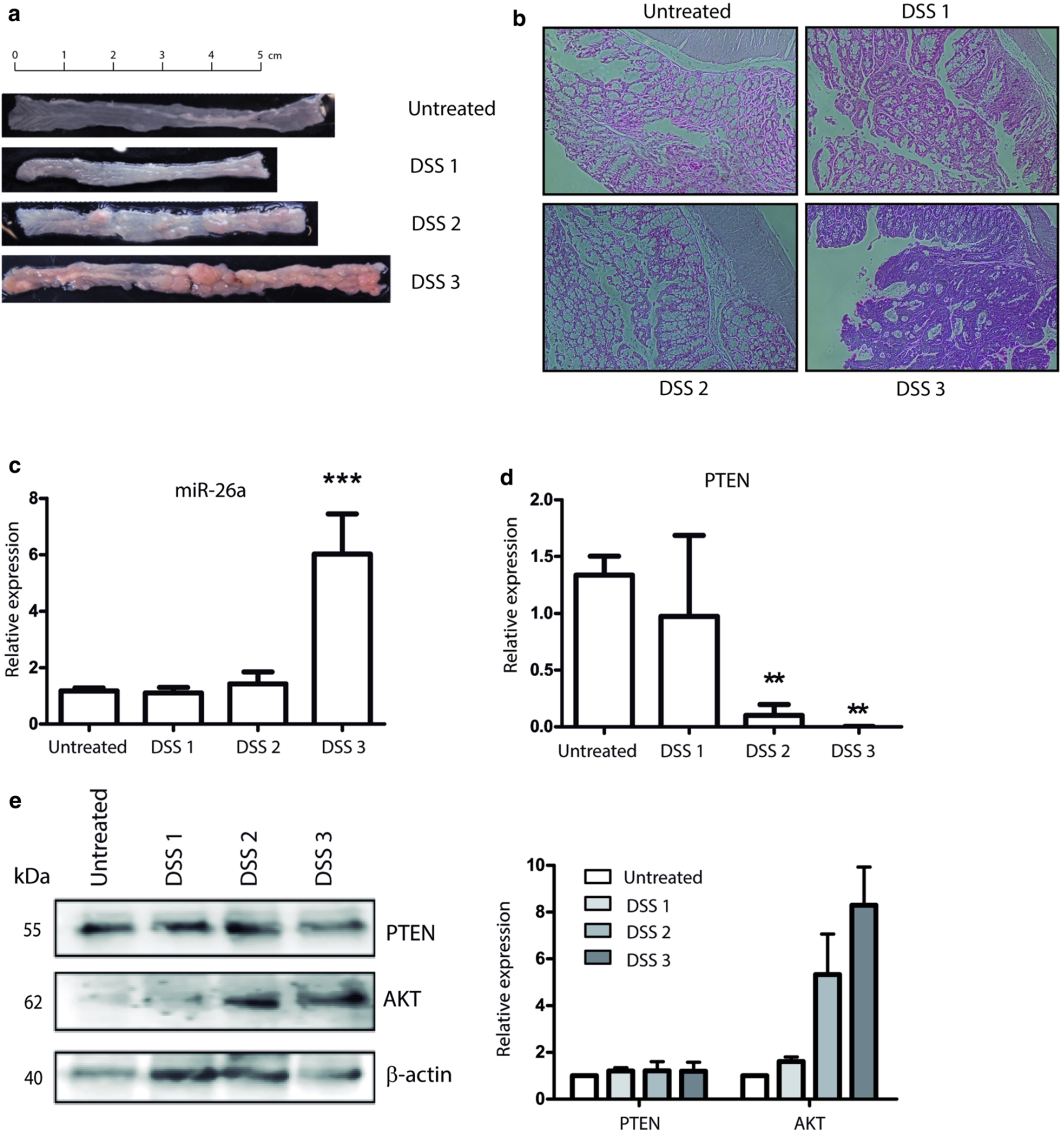
PTEN was downregulated in AOM/DSS induced-CRC mouse model. **a** Macroscopic inspection of the large bowel after each DSS administration. **b** Representative histological analysis of colon mucosa after each DSS administration staining with Hematoxilyn and Eosine, ×100 amplification. **c** mir-26a expression through development of CRC-mouse model measured by RT-PCR and was normalized with RNU6. **d** PTEN mRNA and **e** protein levels were measured by RT-PCR and Western Blot, were normalized with GAPDH and b-actin respectively, PTEN was detected after each DSS administration. A healthy, non-treated mouse was used as control

We established a CRC mouse model to study miR-26a and PTEN expression in different stages of CRC development. All experimental groups received a single dose of Azoxymethane (AOM) and three Dextran Sulfate Sodium (DSS), every third week to generate progressive tumor development. The mean weight of the three animals in each group decreased with each DSS administration and the presence of tumors was detected after the third DSS administration (Fig. 6a). Histological analysis confirmed these results, revealing a generalized loss of epithelial morphology throughout the DSS cycles. After the second DSS administration, we observed chronic intestinal inflammation and slight crypt distortion with epithelial hyperplasia and after the third DSS dose, formation of adenomas composed of tubular and villous structures lined by epithelium with high grade of dysplasia (Fig. 6b). A thorough analysis of this tumors was published in a recent study from our group [20].

Expression of miR-26a and the PTEN messenger were measured after each DSS administration and we found that miR-26a expression levels remained unchanged in first DSS cycle, showed slight increase in second DSS cycle, and increased sixfold by the final DSS cycle (Fig. 6c). PTEN mRNA expression showed a gradual decrease from the first DSS cycle to the third one, in which it was undetectable (Fig. 6d). These results revealed an inverse correlation between miR-26a expression and PTEN mRNA. Conversely, the PTEN protein remained unchanged after the first and second DSS cycles and decreased only slightly after the third DSS cycle. The downstream effector regulated by PTEN activity, AKT, showed a gradual increase expression at the protein level from the first DSS cycle to the end of mouse model, suggesting that a slight decrease in PTEN allows an increase in the expression of AKT in vivo (Fig. 6e). We concluded that miR-26a targets the PTEN mRNA causing its degradation in our mouse CRC model.

Overall, our data showed that mir-26a had a key role CRC development, since it downregulated PTEN and thus enhanced cell proliferation and migration.

## Discussion

**Fig. 7:**
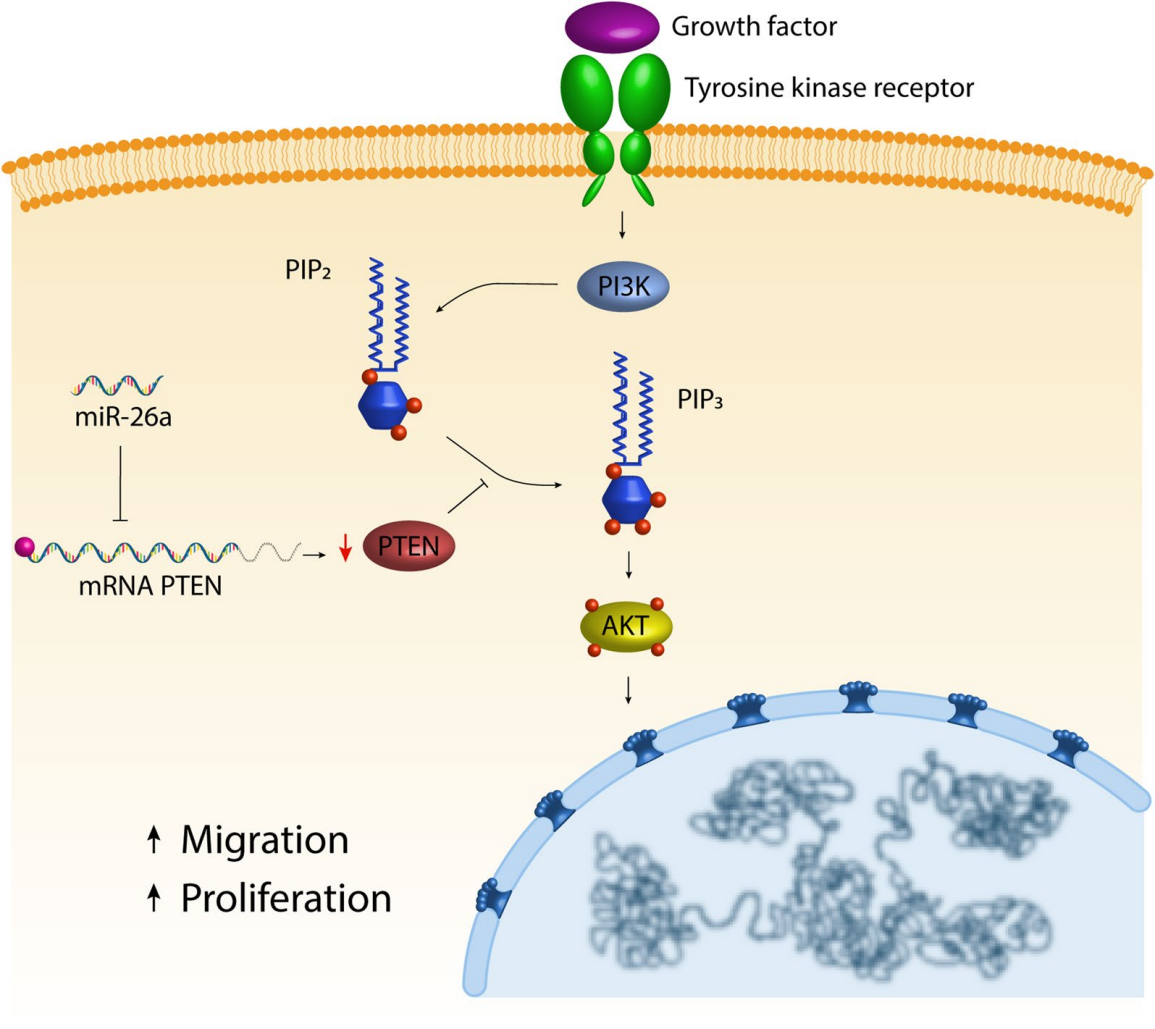
mir-26a is involved in proliferation and migration via direct regulation of PTEN-pAKT

In this study, we found that mir-26a was overexpressed in CRC tissues, CRC-derived cell lines, and in samples listed in the TCGA database; furthermore, we found that mir-26a also was overexpressed in a CRC mouse model and that, when overexpressed in CRC-derive cell lines, it maintains proliferation and enhance migration via direct regulation of PTEN; moreover, mir-26a affected phosphorylation levels of AKT that is an effector of PTEN-PI3K pathway (Fig. 7). The importance of negative regulation of PTEN exerted by miR-26 resides in its phosphatase activity, thus its absence would mean more phosphorylation of its targets [22]. We were able to show (Fig. 4b) that pAKT level is increased when cells were treated with the miR-26 mimetic (i.e. lower PTEN levels lead to lowered phosphatase activity which leads to higher detection of pAKT) and decreased when miR-26 was abolished with the anti-miRNA (i.e. higher PTEN levels lead to increased phosphatase activity leading to lower pAKT detection). Neither mir-26a or PTEN is known to affect AKT expression, therefore its levels remained expectedly unchanged.

PTEN has important role as a negative regulator of survival signaling and metastasis. Several studies have demonstrated that loss of PTEN expression contribute to CRC development and is associated with the migration aggressive capacity [23]. Correspondingly, the PI3K-AKT pathway, which is negatively regulated by PTEN is hyperactive in several cancers [24]. Loss of PTEN function has been characterized in tumors such as glioblastoma, endometrial cancer, non-small cell lung cancer and colorectal cancer among others [25]; yet, the mechanisms that lead to it are still controversial. Recently, Lin and colleagues [26] successfully identified mechanisms such as point mutations and promoter hypermethylation in CRC patient samples with loss of PTEN expression, but failed to identify a mechanism for more than 50% of them. Another study showed that hypermethilation of PTEN promotor occurs only in around 20% of colorectal tumors [2] indicating that other epigenetic mechanisms involved in the negative regulation of PTEN are unclear. Concurrently, several groups have found that the PTEN mRNA is subject to tight miRNA regulation, for instance, it is downregulated by miR-4534 in prostate cancer [27], and miR-29a in breast cancer [28]. Huse and colleagues were able to show that miR-26 targets and induces the degradation of PTEN in high grade glioma tumors [7] and Liu in 2012 demonstrated that mir-26a overexpression enhances migration and invasion process through wound healing and invasion chamber assays respectively, in lung cancer cells by targeting PTEN [29]. But the evidence from lung and colorectal cancer is most interesting: In lung cancer, PTEN is downregulated also by miR-21 [30], miR-205 [31], and miR-92a [32]; while in colorectal cancer, it is regulated by miR-21 [33], miR-92a [34] and miR-106b [35]. To our knowledge, we show for the first-time a negative regulation of PTEN exerted by mir-26a in colorectal cancer.

We found the lowest mRNA and protein expression in SW620 cell line, compared to the other lines used in this study. Interestingly, there was an inverse correlation as this same cell line showed the highest mir-26a levels. HCT116 cell line represents a primary tumor in early stage, in this cell line we did not observe a correlation between mir-26a and PTEN, suggesting another mechanism on PTEN regulation. There are other miRNAs that only are transcribed in primary stages, such as mir-32, mir-200c and mir-221/222. These miRNAs are highly expressed in HCT116, HT-29 [36] and Dukes’ A CRC samples (where HT-29 cells and Dukes’ A samples represent a primary tumor stage) and downregulated in SW480, SW620 and Dukes’ D CRC samples. Therefore, PTEN is regulated by these miRNAs and their overexpression is caused by oncogenic K-RAS mutation, a key event in early CRC development [37]. On the other hand in the cell lines SW480 and SW620 which represent the most advanced stage our data suggested that the mir-26a-PTEN regulation takes place in that stage. This fact could explain the variations in the PTEN expression after mimic and inhibitor transfection in these lines [38]. Our findings complement this data, proving that PTEN is regulated by miR-26a as well. So, thus far, nine different miRNAs have been found to regulate PTEN in CRC: miR-21, miR-92a, miR-106b, mir-32, mir-200c, mir-221/222 and miR-26a. This highlights the importance of PTEN downregulation as a means to achieve cell over-proliferation, and constitutes a plausible candidate for the missing mechanism in the aforementioned work [26].

Chronic inflammatory process is a key event in colorectal CRC development, due to activated inflammatory cells produce reactive oxygen species and reactive nitrogen intermediates which be able to induce DNA damage and mutations. Also, in CRC has been observed immune cells enhance cytokine production and growth factors, causing oxidative damage and epigenetic silencing of tumor suppressor genes [39]. In our results, we noticed that mir-26a expression is slightly higher in Cronh´s disease than healthy tissue, however this result is not statistically significant. Moreover, levels of mir-26a has been found overexpressed in inactive colonic mucosa of patients with ulcerative colitis and Cronh´s disease and it has been considered as a crucial player in these diseases and miR-26 can be used as good diagnostic biomarker [40].

Besides, miR-26a is an important pro-oncogenic regulator on its own, its downregulation impacts several processes that promote the establishment of tumoral phenotype, such as cell proliferation, cellular senescence, cell migration and metastasis [41]. Particularly in CRC, it has been reported to target PDHX [42], Rb1 [14], and, as per the data that we present in this work, PTEN. It has also been demonstrated that PTEN downregulation enhances epithelial-mesenchymal transition by a direct Wnt/B-catenin pathway activation in CRC [43]. Strikingly, GSK3-B—a negative key regulator of this pathway—is also a mir-26a target [8]. Mir-26a has been described as a crucial factor in the regulation of cell death, having a dual function showing a tissue-specific function. In human oral cancer cells, metformin treatment trigger mir-26a overexpression resulting in apoptosis induction [44]. Another study showed this miRNA have a protective role in ethanol-induced acute liver injury through enhancing autophagy by means of regulating DUSP4 and DSP5, two MAPKs inhibitors [45]. Moreover, in our results downregulation of mir-26a significantly reduced cell proliferation, it could be due to lack of mir-26a induces cell death. In hepatocellular carcinoma, overexpression of mir-26a/b inhibits autophagy induced by doxorubicin treatment through regulate ULK1 expression and also induces apoptosis to enhance cell chemosensitivity; On the other hand, low level of mir-26a confers chemoresistance via autophagy induction when cells were treated with doxorubicin [46]. To our knowledge the role of miR-26 related to cell death or cell cycle arrest in CRC deserves new studies, butthis growing body of evidence shows how important a regulator is miR-26a in colorectal cancer development: so far it is known to play a role in cancer cell metabolism, cell migration, and cell proliferation. Then, the question arises as to whether there are more miR-26a targets and how they interact with each other toward the generation and/or maintenance of the tumoral phenotype in CRC.

## Conclusions

Overall our data suggested that mir-26a could be used as a biomarker of tumor development in CRC patients, however more studies must be conducted to stablish its clinical role. Future studies will both validate these interactions in patient cohorts to establish miR-26a as a CRC diagnostic marker, and probably find further miR-26a targets to elucidate a complete picture of the miR-26a regulation network that drives CRC development.
